# Organohalide respiration by a *Desulforhopalus*-dominated community

**DOI:** 10.1093/ismejo/wrag007

**Published:** 2026-01-26

**Authors:** Chen Zhang, Siavash Atashgahi, Tom N P Bosma, Hauke Smidt

**Affiliations:** Laboratory of Microbiology, Wageningen University & Research, Stippeneng 4, 6708 WE Wageningen, The Netherlands; Wenzhou Institute, University of Chinese Academy of Sciences, 325001 Wenzhou, China; Laboratory of Microbiology, Wageningen University & Research, Stippeneng 4, 6708 WE Wageningen, The Netherlands; AB Mauri, Oude Kerkstraat 55, 4878 AK Etten-Leur, The Netherlands; Laboratory of Microbiology, Wageningen University & Research, Stippeneng 4, 6708 WE Wageningen, The Netherlands; Deltares, Daltonlaan 600, 3484 BK Utrecht, The Netherlands; Laboratory of Microbiology, Wageningen University & Research, Stippeneng 4, 6708 WE Wageningen, The Netherlands

**Keywords:** organohalide-respiring bacteria, reductive dehalogenase, 2,6-dibromophenol debromination, *Desulforhopalus*, acetylene inhibition, genome analyses

## Abstract

Marine sediments harbor diverse organohalide-respiring bacteria (OHRB), but their functional roles and metabolic interactions remains poorly understood. To investigate these interactions, we obtained and characterized a debrominating consortium from Aarhus Bay marine sediments. The consortium transformed 2,6-dibromophenol (2,6-DBP) to phenol under sulfate-reducing conditions, with bacterial growth demonstrating respiratory energy conservation. Metagenomic analysis and binning revealed five new species-level populations (>85% complete, <3% contaminated) dominated by *Desulforhopalus* (bin.5). Critically, bin.5 encodes a thiolytic tetrachloro-p-hydroquinone reductive dehalogenase (RDase), previously characterized only in aerobic bacteria, representing evidence of this enzyme functioning in a strictly anaerobic sulfate-reducing bacterium. Two additional populations (*Desulfoplanes* bin.3 and *Marinifilaceae* bin.4) encoded two and one putative respiratory corrinoid-dependent RDase, respectively. Transcription of all four RDase genes was rapidly induced upon 2,6-DBP addition, indicating multi-population response. Acetylene inhibited debromination post-transcriptionally without affecting RDase gene transcription, or sulfate metabolism, confirming RDase-mediated catalysis. Genome analysis indicated bin.5 encodes a near-complete vitamin B_12_ biosynthesis pathway (lacking only *cbiJ*, which can be bypassed through alternative reductases), consistent with debromination activity independent of exogenous B_12_ addition. Comparative genomics identified *Marinifilum* and *Ancylomarina* as candidate OHRB taxa, substantially expanding known phylogenetic diversity of marine organohalide respirers. This work reveals previously unrecognized biochemical versatility in anaerobic dehalogenation and demonstrates metabolic self-sufficiency enabling organohalide respiration in oligotrophic marine sediments.

## Introduction

Metagenomic surveys of marine sediments from Aarhus Bay have revealed diverse reductive dehalogenase (RDase)-encoding genes and corresponding transcripts, suggesting active organohalide respiration (OHR) in these nutrient-limited environments [1–3]. Geochemical profiles showing elevated Br^−^/Cl^−^ ratios with depth suggest prevalent reductive debromination [3], which was validated through laboratory enrichment cultures that dehalogenated tetrachloroethene (PCE), bromophenols, dibromobenzenes, and triiodophenol [1]. Genome-resolved metatranscriptomic analyses identified diverse novel putative organohalide respiring bacteria (OHRB) with PCE-induced RDase expression, particularly at station M5, characterized by suboxic to anoxic sediments with active sulfate reduction and limited oxygen penetration [4–6]. Yet the specific metabolic roles and interactions of these OHRB remain unclear [2]. Understanding these interactions is crucial for elucidating biogeochemical halogen cycling in marine sediments and the ecological strategies enabling microbial dehalogenation in nutrient-limited environments.

Reductive dehalogenation is catalyzed by two biochemically distinct RDases types: (1) respiratory, corrinoid-dependent RDases in anaerobic OHRB that couple dehalogenation to energy conservation [7, 8], and (2) non-respiratory, glutathione-dependent thiolytic tetrachloro-p-hydroquinone (TPh)-RDases primarily characterized in aerobic bacteria [9–11]. Respiratory RDases feature conserved functional motifs, including twin-arginine translocation signal peptides, and iron–sulfur clusters for electron transfer [8, 12–14], while TPh-RDases belong to the glutathione-S-transferase superfamily with conserved cysteine and serine catalytic residues [15]. Although TPh-RDases require reducing conditions for catalysis, they have been characterized exclusively in aerobic bacteria [10, 11], raising questions about their potential function in strict anaerobes. Our previous metagenomic analysis identified a TPh-RDase gene in a *Desulforhopalus*-affiliated metagenome-assembled genome (MAG) from Aarhus Bay sediments with expression induced during PCE dehalogenation [1, 2]. This raised fundamental questions about TPh-RDase function under anoxic conditions and potential metabolic interactions with respiratory RDases-encoding populations.

Respiratory RDases require corrinoid (vitamin B_12_) cofactors for activity. Most characterized OHRB, including *Dehalococcoides* species, lack de novo B_12_ biosynthesis capacity and depend on external corrinoid supplementation [16–20]. In contrast, some OHRB such as *Sulfurospirillum multivorans* possess complete biosynthesis pathways, enabling corrinoid-independent dehalogenation [21]. A recent defined coculture study of *Dehalococcoides mccartyi* strain 195 and *S. multivorans* achieved threefold faster complete PCE dehalogenation to ethene compared to *D. mccartyi* monocultures without added corrinoids, demonstrating metabolic complementation in OHR communities [22]. However, bottom-up construction of defined consortia remains challenging due to limited availability of cultured isolates and incomplete understanding of metabolic interactions. Conversely, top-down isolation from natural communities may reveal functional consortia with self-sufficient metabolic capacities.

To address knowledge gaps and functionally characterize the previously identified TPh-RDase-encoding *Desulforhopalus* population, we obtained and further characterized a B_12_-independent debrominating consortium from Aarhus Bay sediments. Genome-resolved metagenomic analysis revealed five novel species-level populations encoding both respiratory and non-respiratory RDases, with the dominant *Desulforhopalus* population (bin.5) possessing a nearly complete B_12_ biosynthesis pathway [23]. While the five assembled bins dominated the consortium, 16S rRNA gene profiling revealed minor populations including *Klebsiella*, *Myroides*, *Mycoplasma*, and *Endomicrobium*. This work provides evidence for TPh-RDase function in a strictly anaerobic bacterium, elucidates metabolic self-sufficiency enabling OHR in oligotrophic marine environments, and expands the known phylogenetic diversity of marine OHRB to include *Marinifilum* and *Ancylomarina*.

## Materials and methods

### Chemicals

Halogenated compounds including PCE, 2,6-dibromophenol (2,6-DBP), 2,4-DBP, 2,4–6-DBP, 1,4-dibromobenzene (1,4-DBB), 1,2-DBB, 1,3-DBB, 1,2,4-tribromobenzene (1,2,4-TBB), 2,6-dichlorophenol (2,6-DCP), 2,4-DCP, 2,4,6-TCP, 1,4-dichlorobenzene (1,4-DCB), 1,2-DCB, 1,3-DCB, 1,2,4-TCB, benzene, 2,4,6-triiodiphenol (2,4,6-TIP), 2,4-DIP, 2,6-DIP, 2-IP, and 4-IP were purchased from Sigma-Aldrich (St. Louis, MO, USA) with purity ≥98%. Acetylene gas (99.6%) used for inhibition experiments was obtained from Linde Gas Benelux (Schiedam, The Netherlands). Stock solutions of sodium sulfate (0.5 M), sulfite (0.5 M), thiosulfate (0.5 M), nitrate (0.5 M), formate (0.5 M), acetate (0.25 M), pyruvate (0.5 M), and lactate (0.5 M) were prepared from analytical grade reagents (≥99%, Sigma-Aldrich) dissolved in ultrapure water and filter sterilized (syringe filter, 0.2 μm, mdimembrane, Ambala Cantt, India). Salts for marine medium preparation (NaCl, MgCl₂·6H₂O, KCl, NH₄Cl, CaCl₂·2H₂O, K₂HPO₄, NaHCO₃) were of analytical grade (≥99%, Sigma-Aldrich). Na₂S·9H₂O (analytical grade, ≥98%, Sigma-Aldrich) and Resazurin (Sigma-Aldrich) were used as reducing agent and redox indicator, respectively. All other chemicals were of analytical grade (≥95%).

### Consortium isolation and cultivation

Marine medium was prepared for cultivation as previously described [21, 22, 24]. Briefly, the basal medium contained (per liter): NaCl (20 g), MgCl₂·6H₂O (3 g), KCl (0.5 g), NH₄Cl (0.3 g), CaCl₂·2H₂O (0.15 g), K₂HPO₄ (0.2 g), NaHCO₃ (1 g), trace element solution (1 mL), and vitamin solution (1 mL) including vitamin B₁₂ (0.025 mg/L, ~18.5 nM). The medium had an initial pH of ~7.2–7.4 after autoclaving and equilibration with N₂/CO₂ (80:20%, 140 KPa) headspace gas. The final growth medium was composed of 50 mL anoxic marine medium, Na_2_S·9H_2_O (0.48 g/L, 2 mM) serving as the reducing agent, and Resazurin (0.005 g/L) as the redox indicator. The headspace of culture bottles was exchanged with N_2_/CO_2_ (80: 20%, 140 KPa), and bottles were sealed with Teflon-coated butyl rubber septa and aluminum crimp caps (GRACE, MD, USA). pH was not actively monitored during incubation but was expected to remain circumneutral (pH 6.8–7.5) due to the bicarbonate buffering system and continuous CO₂ equilibration from the headspace. For B₁₂-independent culture generation and growth experiments, the vitamin solution was prepared without vitamin B₁₂ supplementation, while all other medium components remained identical to ensure that any observed growth and debromination activity was independent of external cobalamin. Slant tubes contained 5 ml marine medium with 0.8% low-melting point agarose (Sigma-Aldrich) and were incubated in the dark at 20°C.

The original marine sediment samples were collected from station M5, Aarhus Bay, Denmark (56.103333°N, 10.457833°E; water depth 28 m) from sediment depth 3–35 cm as previously described [2]. This depth interval represents suboxic to anoxic sediment layers below the bioturbated zone (0–3 cm), characterized by shallow oxygen penetration (1–5 mm), active sulfate reduction (sulfate 15–25 mM in surface sediments, decreasing below 5–10 cm), and increasing sulfide concentrations (<1 μM at surface to 3–5 mM at depth) typical of organic-rich coastal marine sediments [4, 6, 25]. The experiments described here started from a stable, sediment-free PCE-dechlorinating enrichment culture derived from Aarhus Bay sediments. Briefly, the original marine sediment inoculum was transferred twice and then subjected to two consecutive rounds of serial dilution and incubation as detailed in our previous work [1]. From the second dilution series, 200 μL of the third PCE-dehalogenating culture (10^−3^) was transferred to an anoxic slant tube for isolation in the presence of PCE and sulfate, A range of halogenated compounds, including PCE, 2,6-DBP, 2,4-DBP, 2,4–6-DBP, 1,4-DBB, 1,2-DBB, 1,3-DBB, 1,2,4-TBB, 2,6-DCP, 2,4-DCP, 2,4,6-TCP, 1,4-DCB, 1,2-DCB, 1,3-DCB, 1,2,4-TCB, 2,4,6-TIP, 2,4-DIP, 2,6-DIP, 2-IP, and 4-IP, were tested individually as the electron acceptor with lactate as the electron donor and carbon source. To study the growth of the community on 2,6-DBP (200 μM), hydrogen (20 mM) and acetate (5 mM) served as the electron donor and carbon source, respectively. Community growth was monitored by two methods: (i) optical density at 600 nm (OD_600_) for experiments with lactate as electron donor where cell density was sufficiently high for spectrophotometric detection, and (ii) quantitative PCR (qPCR) targeting the bacterial 16S rRNA gene to determine 16S rRNA gene copy numbers per mL for experiments with H₂/acetate where OD_600_ measurements had insufficient sensitivity. For growth experiments with H₂/acetate and 2,6-DBP, samples were collected every 2 days. Acetylene was injected into the bottles and tested as inhibitor for reductive dehalogenation in three biological replicates, with three replicate control bottles without acetylene. Acetylene was added by injecting 3 mL of acetylene gas directly into the headspace of 120 mL serum bottles containing 50 mL culture medium (headspace volume = 70 mL). Based on the injected volume, headspace pressure, and gas–liquid partitioning according to Henry’s law at 20°C (KH ≈ 0.041 M/atm), the aqueous acetylene concentration was estimated at ~1.8 mM. Prior to the growth experiments, actively debrominating culture was washed to remove residual medium components. Specifically, 2.5 ml of actively debrominating culture was transferred three times consecutively (10% vol/vol inoculum) to 48 ml fresh medium supplemented with H₂/acetate and 2,6-DBP as described above. Each washing transfer was performed in triplicate (n = 3 biological replicates). Each transfer was incubated until active debromination was observed, ensuring complete removal of residual lactate and sulfate from the original medium. After these three washing transfers, a fourth transfer was performed for the actual growth experiment using the same medium composition with three biological replicates per treatment. The generation of B_12_-independent cultures followed the same washing procedure: three consecutive transfers to fresh medium without B_12_ supplementation in triplicate, with each transfer incubated until active debromination was confirmed, followed by a fourth transfer for the actual experimental measurements with three biological replicates. This procedure ensured complete removal of any residual B_12_ from the original medium. For metagenomic sequencing and community composition analysis, biomass was harvested after 5 days of incubation when ~90% of the 2,6-DBP had been debrominated, representing late exponential to early stationary growth phase with active debromination occurring.

### Analytical methods

PCE, trichloroethene (TCE), cis-dichloroethene (cDCE), trans-dichloroethene (tDCE), ethene and acetylene were analyzed by gas chromatography combined with mass spectrometry (GC–MS) with an Rt-Q-BOND column (Restek, PA, USA) and DSQ-MS (Thermo Fisher Scientific). Hydrogen gas was detected by Compact GC (Global Analyzer Solutions, Breda, The Netherlands) with a pulsed discharge ionization detector (GC-PDD). Thermo Scientific Accela High-Performance Liquid Chromatography (HPLC) system installed with an Agilent Poroshell 120 EC-C18 column and a UV/Vis detector was used to measure halogenated phenols, and benzenes, benzene, and phenol. Organic acids, including lactate, propionate, acetate and formate, were measured by SHIMADZU LC2030 PLUS coupled with a Shodex SUGAR Series SH1821 column. Sulfate, sulfite, thiosulfate and nitrate were measured by using the Thermo Scientific Dionex ICS-2100 Ion Chromatography System (Dionex ICS-2100). Sulfide was analyzed photometrically using methylene blue as described previously [24, 26, 27].

### DNA and RNA extraction and reverse-transcriptase quantitative PCR (RT-qPCR)

The cultures were first centrifuged at 10 000 × g for 5 min, the supernatant was discarded, and the pellets were then washed three times with 200 μL TE buffer (pH = 7.0, 4°C) on ice to remove any residual medium components that might interfere with downstream DNA and RNA extraction. For genomic DNA extraction, the MasterPure Gram positive DNA purification Kit (Epicenter, WI, USA) was used, following manufacturer’s instructions. For RNA isolation, prior to the sample collection, cultures in eighteen bottles were grown with additional lactate (15 mM) and sulfate (20 mM) to exponential phase for 72 h after transfer without any additional 2,6-DBP. Nine bottles were collected as the 0 h samples (n = 9). From the remaining nine bottles, three were kept as before without any injection (n = 3), three replicate cultures were injected with 200 μM 2,6-DBP (n = 3), and the last three were injected with 200 μM 2,6-DBP and 1.8 mM acetylene (n = 3), and cells were collected after 0.5 h and washed as described above. Collected biomass was mixed with 0.4 ml cold TE buffer (4 μl *2*-mercaptoethanol), and 0.5 ml TRIzol reagent (Thermo Fisher Scientific) was added, followed by bead-beating for 3 min (3 times, 1 min per time with cooling on ice in between) at speed 5.5 (FastPrep-24 5G, MP biomedicals, Irvine, CA, USA). After bead-beating, 200 μl of UltraPure phenol: chloroform: isoamyl alcohol (25:24:1 vol/vol/vol; Thermo Fisher Scientific) was added and mixed by vortex. Then, the separated aqueous phase containing RNA was transferred to an RNeasy column (Qiagen, Venlo, The Netherlands) for purification followed by DNase I (Roche, Almere, The Netherlands) treatment to remove residual DNA. In order to check for purity of the obtained culture, a near-full length fragment of the bacterial 16S ribosomal RNA (rRNA) gene was amplified using universal bacterial primers 27F/1492R (Table 1) and subjected to Sanger sequencing as previously described [28]. Bacterial 16S rRNA gene-targeted qPCR was used for assessing the growth of the microbial community via the increase of 16S rRNA gene copy numbers using the general primers Eub341F/Eub534R (Table 1). qPCR was the primary method for growth quantification in experiments where cell density was too low for reliable OD_600_ measurements, providing accurate abundance expressed as bacterial 16S rRNA gene copies per mL of culture. RT-qPCR was introduced to measure the relative expression of RDase genes by using the One Step PrimeScript RT-PCR Kit (Perfect Real Time) (Takara Bio, Saint-Germain-en-Laye, Germany). Primers targeting RDase genes were designed using the NCBI primer design tool with the following parameters: Expected PCR product sizes ranged from 75 bp to 150 bp and melting temperatures from 57°C to 60°C (Table 1). RT-qPCR targeting the bacterial 16S rRNA gene was used for normalization of RDase gene expression. Relative expression levels were calculated using the ΔΔCt method, where Ct values of RDase genes were first normalized to 16S rRNA gene (ΔCt), and then fold changes were calculated relative to the control condition (0 h, no 2,6-DBP addition) to obtain ΔΔCt values. This approach accounts for differences in total RNA content and cell numbers across samples, allowing comparison of RDase expression levels between different treatment conditions.

**Table 1:** Primers used in this study for (RT)-quantitative PCR.

**Target**	**Name**	**Oligonucleotide sequence (5′–3′)**	**Purpose and (RT)-qPCR programs**
Bacterial 16S rRNA gene	Eub341F Eub534R	CCTACGGGAGGCAGCAG ATTACCGCGGCTGCTGGC	qPCR bacterial abundance
			
Bacterial 16S rRNA gene	27F 1492R	AGAGTTTGATCMTGGCTCAG TACGGYTACCTTGTTACGACTT	Universal for Bacteria Sanger Sequencing
			
bin.3-RDase1	B3RD1-919F B3RD1-1044R	GTCATGTCCGGAATGGGTGA GTCTATGGGCTTGGTCTCGG	qPCR RDase genes Stage1: Reverse transcription 42°C 5 min 95°C 10 s Stage2: PCR reaction 95°C 5 s 60°C 20 s 40 Cycles
		
bin.3-RDase2	B3RD2-479F B3RD2-598R	CCAACACTGATGCCGCAAAT TGTGCTGGGTTTCGCTGTAT	
		
bin.4-RDase	B4RD-734F B4RD-864R	CCGACTGGAGCGATTTTCCT CATGGCAGAATAACCGGCAC	
		
bin.5-TPh-RDase	B5TRD-282F B5TRD-430R	CGGATCGCTTCGTCCTGAAT CCATCTGCTCAGTTGTTCGC	
		

### Metagenome sequencing and analyses

Metagenome sequence data were generated by Illumina paired-end short read (PE150, Novaseq6000 System, Novogene) and PacBio long read sequencing (Sequel, Novogene). The raw paired-end Illumina and PacBio reads were first examined using the quality check module “read_qc” of MetaWRAP [29]. Low-quality reads as well as human contaminant reads were removed. The raw PacBio long-read data were combined to improve the quality of assembly following OPERA-MS (v0.8.3) [30]. Considering the improved features of the hybrid assembly in comparison to the short-read assembly, including contigs’ N50, maximum contig size and number of contigs, the follow-up analyses were based on the hybrid assembly. Kraken2 (v2.1.1) classifies individual metagenomic sequencing reads by matching k-mers to a reference database, SILVA database version 138. Thus, the relative abundance was derived from the proportion of reads assigned to each taxon out of the total reads of the 16S rRNA gene [31]. Relative abundances derived from 16S rRNA gene sequences were not corrected for genomic gene copy number variation, which may introduce bias in proportional abundance estimates. The resulting hybrid assembly was binned and refined using the MetaWRAP (v1.3) modules, “Binning module” combining metabat2, maxbin2 and concoct with the cut-off set at >50% completeness and < 10% contamination, and “Bin_refinement” to improve the bin set. The abundance of the refined bins was estimated using the “quant_bins” module in MetaWRAP (v1.3), which uses Salmon to index the entire metagenomic assembly. Sequencing reads from samples were mapped back to the hybrid assembly. The resulting coverage estimates were used to calculate the abundance of each contig in each sample. The abundance of each bin was subsequently determined as the length-weighted average of the abundances of its constituent contigs. Bins were reassembled, taxonomically classified and annotated via the “classify_wf” workflow of GTDB-Tk (v1.5.0) using by-default parameters and the “prokka” module of MetaWRAP, respectively [32].

### Phylogenetic analysis of bins and inference of metabolic pathways

Five bins were assembled, and their classification by GTDB-Tk revealed that all of them were potentially new species as their average nucleotide identity (ANI) with entries in GTDB (v207) was lower than 95%. Fifty-two representative genomes most closely related to the bins were extracted from the GTDB database (v207) and were included for phylogenetic analysis by GToTree (v1.5.50). Metabolic traits associated with reductive dehalogenation were also inferred, including the metabolic genes encoding RDase (*rdh*), TPh-RDase (TPh*-rdh*), haloacid dehalogenase (*hdh*), pathways involved in the metabolism of sulfate, nitrate, hydrogen, nitrogen, acetylene, Wood-Ljundahl pathway (WLP), de novo B_12_ biosynthesis and B_12_ transporters. To this end, MagicLamp (v1.0) was used with the retrieved COG numbers of metabolic genes from the .gff format of bins and their related representative genomes to infer the metabolic potential of the consortium. The constructed phylogenetic tree was visualized and modified by ggtree [33].

## Results

### Debrominating potential of the obtained consortium

**Figure 1:**
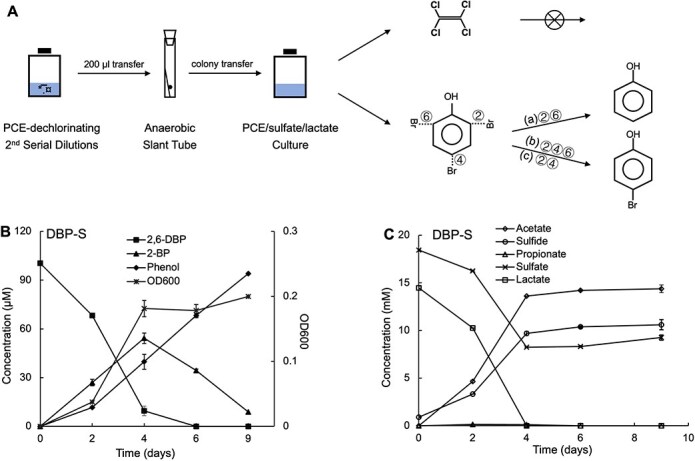
Schematic diagram of colony isolation and its reductive debromination of 2,6-dibromophenol (2,6-DBP) in the presence of sulfate (DBP-S). The mother bottle (10^−3^) was selected from the second serial dilution in the presence of sulfate as previously described [2]. The colony-derived culture could completely debrominate 2,6-DBP to phenol, whereas 2,4,6- or 2,4- brominated phenols were transformed into 4-bromophenol (4-BP) (A). Debromination of 2,6-DBP to phenol (B) with the formation of 2-bromopenol (2-BP) as the intermediate, together with sulfate reduction to sulfide (C) to support the bacterial growth (OD600). The symbols, ②, ④, and ⑥ represented the bromide positions, while (a), (b), and (c) indicated the 2,6-DBP, 2,4,6-TBP, and 2,4-DBP, respectively. Three replicate bottles were set for the reductive debromination of 2,6-DBP with additional sulfate. Data are presented as mean ± standard deviation (SD). Error bars indicate the SD.

To obtain members of the stable, sediment-free PCE dechlorinating enrichment derived from Aarhus Bay sediment [2], we inoculated a sample of this culture onto an anoxic agar slant (Fig. 1A). We were able to retrieve a single colony that developed after 69 days of incubation. After transfer to liquid medium, the derived consortium exhibited debrominating activity with 2,4,6-DBP and 2,6-TBP, resulting in the production of 4-BP and phenol, respectively. The derived culture was, however, no longer able to dechlorinate PCE (Fig. 1A). To better understand the physiological traits of the colony-derived consortium, different electron donors and carbon sources, such as H_2_/acetate and pyruvate, and various electron acceptors, such as sulfate and nitrate, and particularly a range of different halogenated compounds, were tested (Table 2). In line with the initial results, the culture only displayed debrominating potential rather than dechlorination or deiodination. While the colony-derived consortium was able to utilize sulfate, sulfite, and thiosulfate as electron acceptor, it was not capable of reducing nitrate. The culture was found to completely debrominate 2,6-DBP to phenol after 9 days with the formation of bromophenol as the intermediate (Fig. 1B). The added sulfate served as a competitive electron acceptor and was reduced to sulfide, in which lactate was utilized as the electron donor and carbon source with the production of acetate (Fig. 1C). In line with the observations for the incubation in the presence of sulfate, the reductive debromination also proceeded in the sulfate-free culture (Fig. S1A), in which lactate was consumed fermentatively with the formation of propionate and acetate in a ratio of ~2:1 (Fig. S1B).

**Table 2:** Physiological properties of the obtained consortium.

**Compounds as e-donors and e-acceptors**	**Utilization**
*Compounds used as electron donor and/or carbon source*	
H_2_^a^	+
Formate^a^	+
Acetate	−
Lactate	+
Pyruvate	+
*Fermentative growth on*	
Lactate	+
Pyruvate	+
*Compounds used as electron acceptor*	
Sulfate	+
Sulfite	+
Thiosulfate	+
Nitrate	−
*Halogenated compounds used as electron acceptor* ^b^	
Perchloroethylene (PCE)	−
2,6-Dibromophenol (2,6-DBP); 2,4-DBP; 2,4,6-DBP;	+; +^c^; +^c^;
1,4-Dibromobenzene (1,4-DBB); 1,2-DBB; 1,3-DBB; 1,2,4-TBB	+^d^; −; −; −;
2,6-Dichlorophenol (2,6-DCP); 2,4-DCP; 2,4,6-TCP;	-; −; −
1,4-Dichlorobenzene (1,4-DCB); 1,2-DCB; 1,3-DCB; 1,2,4-TCB;	-; −; −; −;
2,4,6-Triiodophenol (2,4,6-TIP); 2,4-DIP; 2,6-DIP; 2-IP; 4-IP;	-; −; −; −; −;

a Used as the electron donor only in the presence of acetate as carbon source.

b Tested with lactate as electron donor and carbon source.

c 4-bromophenol, not phenol, was formed as the final product.

d Bromobenzene, not benzene, was formed as the final product.

To assess the composition of the colony-derived consortium, Sanger sequencing of a PCR product obtained using universal bacterial 16S rRNA gene-targeted primers 27F / 1492R was attempted. However, the resulting sequence could not be resolved, confirming that the consortium comprised multiple species rather than a pure isolate. Scanning electron microscopy (SEM) confirmed the presence of diverse morphologies in the community (Fig. S1C), with a rod-shaped cell-type being predominant.

### Bacterial growth supported by reductive debromination of 2,6-DBP

**Figure 2:**
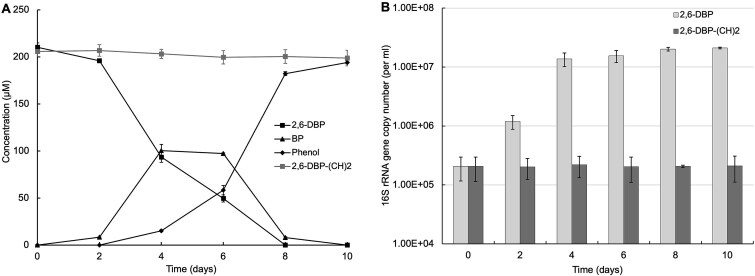
Reductive debromination (A) and growth of the culture (B). H2/acetate served as the electron donor and carbon source for 2,6-DBP debromination, and acetylene added to the negative control (gray line) inhibited 2,6-DBP debromination (A). Quantitative PCR targeting the bacterial 16S rRNA gene was used to assess the cell growth with and without acetylene added (B). (CH)2: Acetylene; three replicate bottles were set for the experiment, and data are presented as mean ± standard deviation (SD). Error bars indicate the SD.

Reductive dehalogenation has previously been shown to be catalyzed by respiratory corrinoid-dependent RDase or by non-respiratory glutathione-dependent TPh-RDase [7, 9, 11, 34]. Reductive dehalogenation enabled by respiratory RDase as the terminal electron accepting process contributes to respiratory energy conservation for bacterial growth [7, 35]. Acetylene has been shown to inhibit reductive dehalogenation by *Dehalococcoides* at a concentration well above 0.4 mM [36, 37]. To confirm its specificity in our consortium, we tested acetylene’s effects on multiple metabolic processes (Fig. S2). In cultures without acetylene, using H_2_/acetate as e-donor and 2,6-DBP as terminal electron acceptor, the bacteria grew from 2.06 (± 0.90) × 10^5^ to 1.59 (± 0.16) × 10^7^ 16S rRNA gene copies / ml after the complete debromination of 200 μM 2,6-DBP in 10 days (Fig. 2). In contrast, in the presence of acetylene (1.8 mM), both growth and debromination were completely inhibited (Fig. 2A), while sulfate reduction and lactate metabolism remained unaffected (Fig. S2). This finding indicated that acetylene specifically inhibits RDase-mediated debromination and the associated respiratory energy conservation, without general cytotoxicity to the consortium.

### *Desulforhopalus*-dominated reductively debrominating consortium

**Figure 3:**
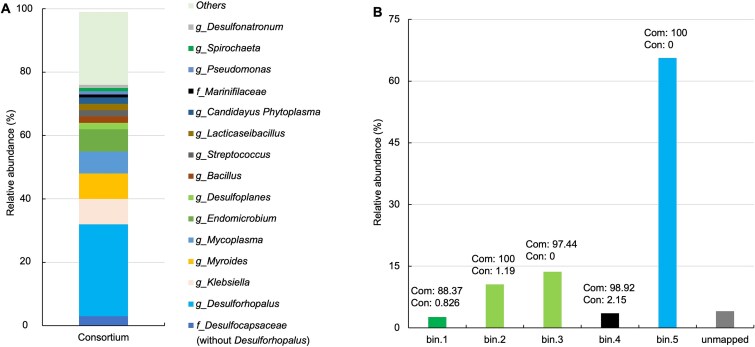
Relative abundance of microbial community members and the assembled bins within the colony-derived consortium. Microbial composition was calculated based on the mapping file generated from the clean raw Illumina reads against the SILVA database version 138 (A). The hybrid assembly was binned into five bins, and the relative abundance of each bin was calculated based on its numbers of reads over the numbers of total reads (B). “Others” represent the assigned taxa with a relative abundance <1% and unassigned taxa; *f*, family; *g*, genus. Com: Completeness (%); con: Contamination (%); unmapped: Reads were not binned.

To further investigate the composition and metabolic potential of the consortium, metagenome sequencing, including short-read and long-read sequencing, was employed. Taxonomic assignment of 16S rRNA gene sequences derived from the metagenome dataset revealed that *Desulforhopalus*, classified into *Desulfobacterota*, accounted for the largest percentage with 29%, followed by populations of *Klebsiella* and *Myroides* with 8% each, and genera *Mycoplasma* (7%) and *Endomicrobium* (7%) (Fig. 3A). In addition, 11% of the sequences were affiliated with the *Bacterodia,* while 10% belonged to *Desulfobacterota* other than *Desulforhopalus*. This overall aligned well with the community analyses of the original PCE-dechlorinating cultures [2]. To accurately decipher the taxonomy and genomic information, short and long metagenome sequence reads were assembled and binned into five genomes (MAGs) that were taxonomically classified using the GTDB-Tk (v1.5.0) “classify_wf” workflow based on the GTDB database (v207) [32]. Bin.1 was found to belong to the genus *Oceanispirochaeta*, bin.2 and bin.3 were affiliated to *Desulfoplanes*, bin.4 to the family *Marinifilaceae*, and bin.5 was affiliated with the genus *Desulforhopalus* (Table 3). All five MAGs were identified as new species based on ANI values with most closely related species being lower than 95%. The reads of the five MAGs accounted for 96.0% of total reads, with bin.5 accounting for 65.6%, bin.3 13.6%, bin.2 10.6%, and bin.4 for 3.6%, leaving only 4.0% of all reads unmapped (Fig. 3B). It is noted that the more quantitative assessment based on metagenomic read mapping is independent of 16S rRNA gene copy number and provides more accurate population abundance estimates. Furthermore, bin.5 was characterized by 100% completeness and 0% contamination according to 120 bacterial single-copy maker genes. In summary, both approaches supported the notion that bin.5, identified as member of *Desulforhopalus*, was the most abundant member of the consortium.

**Table 3:** Information of the assembled bins (MAGs).

**Bins**	**Classification**	**GC (%)**	**N50 (bp)**	**Size (bp)**	**Abundance** ^**a**^	**ANI** ^**b**^ **(%)**
bin.1	*g_Oceanispirochaeta*	43.1	10 840	5 074 027	392.735242	93.85
bin.2	*g_Desulfoplanes*	51.4	199 123	3 841 128	1567.138705	78.11
bin.3	*g_Desulfoplanes*	50.3	87 237	4 114 339	1983.865769	78.14
bin.4	*f_Marinifilaceae*	38.6	43 215	5 488 154	523.39148	N/A^c^
bin.5	*g_Desulforhopalus*	49.3	837 424	3 685 696	9786.929752	76.45

a Abundance: the length-weighted mean contig coverage.

b ANI: average nucleotide identity to the closest reference genome.

c N/A: indicates novel genus without closely related reference genome.

### RDase genes and their expression during reductive debromination

**Figure 4:**
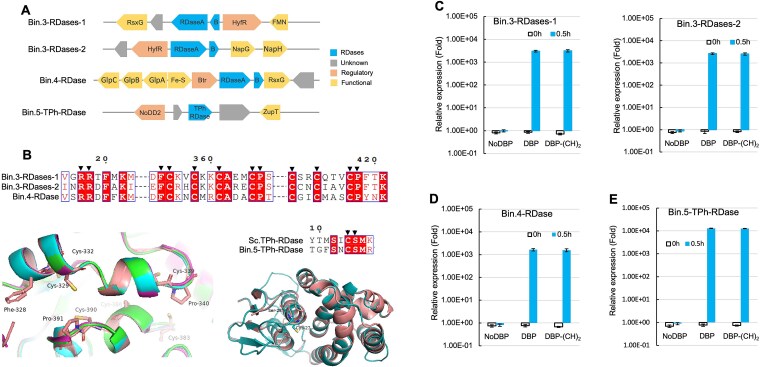
The genomic context, conserved motifs and structures, and expression of RDase genes predicted to encode reductive dehalogenases identified in the bins. RDase genes with the two neighboring genes located up- and downstream (A). Amino acid sequence alignment of conserved motifs of the respiratory RDases of bin.3 and bin.4, and TPh-RDase of bin.5 and *Sphingobium chlorophenolicum*, respectively displayed by EsPript 3.0, and corresponding superimposed structures as generated by PyMol (v.2.3.4) (see below for additional details) (B). Reverse-transcriptase quantitative PCR was used to measure the expression of RDase genes from bin.3 (C) and bin.4 (D), and TPh-RDase gene from bin.5 (E), before (0 h) and 30 min (0.5 h) after addition of 2,6-DBP only, no addition, and addition of 2,6-DBP and acetylene together. Expression values were normalized to 16S rRNA measurements. For the structures shown in B, respiratory RDase (PDB: 5m2g1, in salmon) from *Sulfurospirillum multivorans* was aligned as the best structural template [12]. In the superimposed structure, bin.3-RDase1 is depicted in green, bin.3-RDase2 in cyan and bin.4-RDase in magenta. TPh-RDase (PDB: 7za5.1, in salmon) is shown as dimer as the structural basis for TPh-RDase of bin.5 in teal. FMN: Flavoprotein that can bind the flavin mononucleotide (FMN) as the prosthetic group; RDases: Composed of RDaseA and anchor protein B; HyfR: Transcriptional regulator of formate hydrogenase; RsxG: Electron transporter; NapGH: Electron transporter associated with periplasmic nitrate reductase. GlpABC: Anaerobic glycerol-3-phosphate dehydrogenase complex; Btr: Transcriptional regulator; Fe-S: Ferredoxin with iron–sulfur clusters; TPh-RDase: None respiratory RDase; NoDD2: Regulator for nodulation; ZupT: Zinc transporter. Arrows indicate the conserved sites, in which some were labeled in the superimposed the structure. NoDBP: no 2,6-DBP added; DBP-(CH)2: Both 2,6-DBP and acetylene added. Each treatment in C-E was set with three replicates and values represent mean ± standard deviation (SD). Error bars indicate the SD.

To further examine which of the bins was responsible for the observed reductive debromination, MAGs were annotated, leading to the identification of three genes predicted to encode canonical corrinoid-dependent RDases, two of which were found on bin.3 and one on bin.4. A fourth gene, predicted to code for a thiolytic glutathione-dependent RDase was found on bin.5 (Fig. 4A). No additional RDase-encoding genes were detected in the unbinned fraction of the assembly. BLAST searches against known PCE/TCE reductive dehalogenases (*pceA*, *tceA*) from characterized dechlorinating bacteria revealed no significant homologs in any of the five MAGs, consistent with the loss of PCE dechlorination capability observed in the obtained consortium. Subsequently, the genomic context, i.e. the two neighboring genes upstream and downstream of each RDase gene, was included in the analysis. All three RDase genes encoding the catalytic subunit of respiratory RDases (RdhA) were accompanied by genes predicted to encode the cognate membrane anchor protein (RdhB). In our case, both RDase genes found on bin.3 were accompanied by a gene predicted to code for a formate hydrogenase transcriptional activator, HyfR [38], suggesting both loci originated from the same ancestor. Another transcriptional regulator most closely related to Btr that has been shown to be involved in siderophore bacillibactin production under iron-limiting conditions [39], was found encoded upstream of RDase in bin.4. Finally, upstream of the TPh-RDase encoding gene we observed a gene predicted to code for a transcriptional regulator most similar to NoDD2, regulating nodulation factor production [40]. Although these different regulators have been described with divergent functions, their roles are in general closely related with energy conservation and nutrient acquisition. As to the three predicted corrinoid-dependent RDases, three commonly conserved functional motifs were found, including a twin arginine signal peptide (RR), as well as two iron–sulfur cluster motifs, FCXXCXXCXXXCP and CXXCXXXCP, that were conserved in sequence and structural configuration for binding the iron–sulfur clusters to transfer electrons (Fig. 4B). TPh-RDase was classified into the superfamily of glutathione-S-transferases (GST) that differ from respiratory RDases in sequence and conserved motifs. Sequence alignment of TPh-RDase in bin.5 to that of *Sphingobium chlorophenolicum* revealed that two catalytic site residues, cysteine (C) and serine (S), were conserved in sequence and simulated structure. All four RDases were predicted to be membrane-spanning enzymes, suggesting direct contact with substrates and dehalogenation in the extracytoplasmic space (Fig. S3).

As a next step, we analyzed the transcription of RDase genes using RT-qPCR. This analysis revealed that expression of all four RDase genes was induced several orders of magnitude within 30 min after the addition of 2,6-DBP. More specifically, the relative expression of the TPh-RDase gene increased up to 1.30 (± 0.16) × 10^4^ folds compared to the control to which no organohalide was added. Similarly, expression of genes encoding the respiratory RDases, i.e. RDase-1 and RDase-2 of bin.3, and RDase of bin.4, increased by 3.04 (± 0.29) × 10^3^, 2.63 (± 0.24) × 10^3^, and 1.65 (± 0.20) × 10^3^ folds, respectively (Fig. 4C). We found that the inhibitor acetylene did not affect RDase gene expression, suggesting that inhibition acts post-transcriptionally.

### Comparative genomic analyses of bins and representative genomes of closely-related taxa

**Figure 5:**
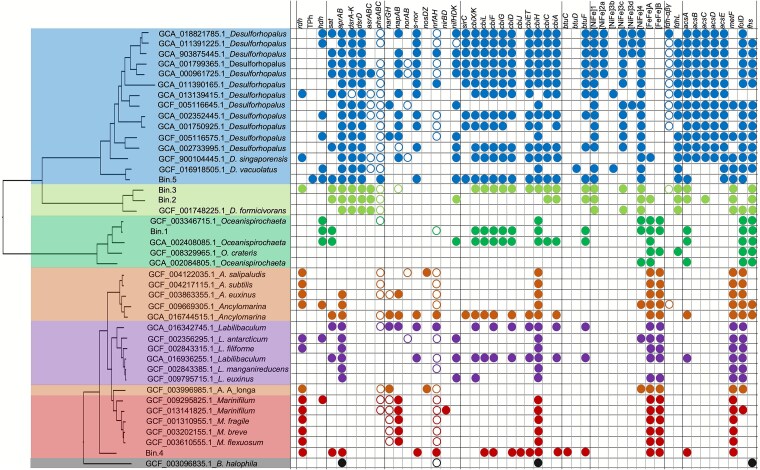
Phylogenies of the assembled bins and most closely related reference genomes, and comparison of their metabolic genes. In addition to the five bins, 52 related representative genomes from the GTDB database (v207) were included for tree construction by GToTree following the default parameters. Filled circles represent encoded functional genes or complete gene clusters. Open circles represent genes for which not the entire cluster was detected. Colors indicate the different phylogenies of bins and their closest relatives. *D. Formicivorans*: *Desulfoplanes formicivorans*; *rdh*: RDase gene; TPh: TPh-RDase gene; *hdh*: Haloacid dehalogenase gene; *sat*, *aprAB*, *dsrA-K*(*ABCTMK*), *dsrD*, *asrABC*, and *phsABC* for sulfate reduction [42]; *narGH* and *napAB* for nitrate reduction [43]; *norAB*, *nirBD*, and *nosDZ* for denitrification [44]; *nrfAH* for ammonification [45]; from *sirC* to *cbiA* for the complete anaerobic de novo corrinoid biosynthesis [23]. *btuFCD* encodes the ABC-type corrinoid transporter [46]; four groups of [Ni-Fe] hydrogenases, 1, 2a, 3b, 3c, 3d, and 4, and two groups of [Fe-Fe] hydrogenases, A and B [47, 48]; *fdh-αβγ*, or *fdhABG*, codes for formate dehydrogenase [49, 50]; *fdhL* codes for NAD^+^-dependent formate dehydrogenase [51, 52]; *acsABCDE*, *folD*, *metF* and *fhs* are critical genes for the wood-Ljungdahl pathway (WLP) [53, 54].

To gain a better understanding of the phylogenies and metabolic traits of bins compared to genomes of closely related organisms, we constructed a phylogenetic tree of bins and associated representative genomes, and subsequently investigated encoded metabolic traits related to the above-mentioned physiological data (Fig. 5). As mentioned above, the five MAGs were classified into four taxonomical groups, in which bin.1 was affiliated with the genus *Oceanispirochaeta*, bin.2 and bin.3 were assigned to the genus *Desulfoplanes*, bin.4 was affiliated to the family *Marinifilaceae*, and bin.5 was assigned to the genus *Desulforhopalus*. The 15 genomes from *Desulforhopalus*, including bin.5, all bear the conserved metabolic genes for sulfate reduction, *de-novo* corrinoid biosynthesis, H_2_ metabolism, and Wood-Ljungdahl pathway (WLP). Whereas bin.5 was predicted to encode a glutathione-dependent TPh-RDase, the genomes of three other *Desulforhopalus* members contained genes predicted to code for corrinoid-dependent respiratory RDases, suggesting that these strains might have OHR potential. Furthermore, whereas most *Desulforhopalus* spp. genomes included in our analysis encoded the complete WLP, two members, *Desulforhopalus vacuolatus* and bin.5 were found to lack the core genes of the WLP, including *acsA*, *acsB* and *acsCD*, coding for carbon monoxide dehydrogenase, acetyl-CoA synthase and corrinoid iron sulfur protein (CFeSP), respectively [41]. The genome of one of the isolates, *D. singaporensis,* encodes all genes for the complete WLP indicating the co-occurrence of OHR potential with the assimilation of C1 compounds. In contrast, bin.1, bin.2, bin.3 and bin.4 and their associated reference genomes were all found to lack the core genes for WLP, suggesting their incapability of one-carbon compound fixation.

B_12_ is the required cofactor for corrinoid-dependent RDases linked with OHR [19]. Based on the analysis of bins, the de novo biosynthesis observed for the colony-derived consortium could be achieved by bin.5 separately, containing the nearly complete gene sets for B_12_ biosynthesis. The *cbiJ* gene, which encodes cobalt-precorrin-6A reductase, was absent from bin.3 (also lacking the *sirC* gene) and bin.5 but present in bin.4. CbiJ catalyzes the NADH-dependent reduction of cobalt-precorrin-6A to cobalt-precorrin-6B in the B_12_ biosynthetic pathway. However, this step can be bypassed or substituted by alternative reductases in some organisms, as demonstrated in marine *Desulfoluna* strains capable of OHR [24]. Therefore, This B_12_-prototrophic phenotype is most likely attributable to bin.5 indicating B_12_ prototrophy at the individual population level rather than requiring consortium-level metabolic complementation. To confirm B_12_ prototrophy, the consortium was cultured with and without vitamin B_12_ supplementation after three consecutive washing transfers. No significant difference in debromination activity or growth was observed between the two conditions (Fig. S4), demonstrating that the consortium does not require external cobalamin for organohalide respiration, consistent with the identified de novo B_12_ biosynthesis capacity.

All six genomes affiliated with the genus *Marinifilum*, including bin.4, were predicted to encode respiratory corrinoid-dependent RDases. Similarly, five out of six genomes affiliated with the closely related genus *Ancylomarina* were predicted to code for corrinoid-dependent RDases. Accordingly, these genera, as a subgroup within the *Marinifilacease* family, are predicted to comprise new OHRB taxa. In addition, all included genomes of these taxa contained genes encoding type A and type B [Fe-Fe] hydrogenases for catalyzing H_2_ metabolism, in contrast to *Desulforhopalus* spp. genomes that were predicted to encode [Ni-Fe] hydrogenases. As outlined above, the critical *cbiH* gene of de novo B_12_ biosynthesis [23], was found conserved among the members of both genera *Ancylomarina* and *Marinifilum* suggesting a symbiotic role in de novo B_12_ biosynthesis. Being closely related to *Ancylomarina* and *Marinifilum*, available genomes of the genus *Labilibaculum* were found to contain similar functional genes, such as genes coding for [Fe-Fe] hydrogenases and CbiH, but only two of the six genomes included here, namely *L. antarcticum* and *L. filiforme*, were predicted to encode corrinoid-dependent RDases.

Physiological characterization of the consortium showed that the community was unable to reduce nitrate as the terminal electron acceptor, which is in line with observed lack of genes encoding membrane-bound and periplasmic nitrate reductase (NarAB and NapAB) in the assembled bins. Similarly, the five bins retrieved from the consortium also were found to lack *norAB*, *nosDZ*, and *nrfAH* genes, indicating their inability of denitrification and ammonification, respectively [44, 45]. Most of the reference genomes from the genera *Desulforhopalus* and *Marinifilum* were found to contain *napAB*, suggesting their potential to reduce nitrate to nitrite. Further, genes encoding the complete nitrogenase complex for nitrogen fixation were found in bin.2 and bin.5, suggesting their capability of fixing molecular nitrogen to produce ammonia in case of N-shortage.

## Discussion

This study advances understanding organohalide respiration in marine environments through three key discoveries. First, we provide evidence that a TPh-RDase, previously characterized exclusively in aerobic bacteria, functions under strictly anoxic conditions in a sulfate-reducing *Desulforhopalus* population, challenging established paradigms about this enzyme class. Second, multiple RDase types (respiratory corrinoid-dependent and non-respiratory glutathione-dependent) were simultaneously induced upon substrate addition, with all populations stably maintained across transfers, suggesting functional coexistence the precise nature (cooperative, competitive, or niche-partitioned) of which requires further investigation. Third, bin.5 encodes a nearly complete vitamin B_12_ biosynthesis pathway (lacking only *cbiJ*, which can be bypassed through alternative reductases as demonstrated in marine *Desulfoluna* strains), consistent with observed B_12_-independent debromination activity. These findings expand understanding of reductive dehalogenase biochemical versatility and metabolic interactions sustaining OHR in nutrient-limited marine sediments.

The consortium was obtained from a stable PCE-dechlorinating enrichment culture containing *Desulfoplanes*, *Desulfobacter*, *Bacillus* and *Desulforhopalus* [2]. In this process, PCE dichlorination capability was lost while 2,6-DBP debromination was retained with *Desulfoplanes* and *Desulforhopalus* populations remaining abundant (Fig. 3), suggesting their role as debrominators rather than dechlorinators. The absence of *pceA*/*tceA* homologs in assembled MAGs provides a genetic basis for this functional shift. The RDase genes in bin.3 and bin.4 represent novel variants with low sequence similarity (< 30%) to those encoding characterized enzymes, suggesting distinct substrate specificities aligning with observed debromination activity. While unbinned populations (4% of reads) could theoretically contribute, no additional RDase genes were detected in unbinned contigs. Strong induction of the four identified RDases upon substrate addition supports that characterized populations are responsible for debromination. Nonetheless, definitive functional confirmation will require strain isolation and physiological characterization.

Bin.5 (*Desulforhopalus*) encodes a TPh-RDase, formerly characterized only in aerobic bacteria [11], suggesting this enzyme can function in strict anaerobes. Sequence alignment and structural modeling confirmed membrane localization (Fig. S3) and conservation of catalytic residues (Fig. 4). In addition, cysteine residues are conserved in both enzyme types but serve different roles: binding B_12_ in corrinoid-dependent RDases versus forming a covalent intermediate with glutathione in TPh-RDases [15, 55].

Acetylene specifically inhibited debromination without affecting sulfate reduction and lactate oxidation, arguing against general metabolic toxicity and supporting targeted inhibition of RDases or their electron transfer partners (Figs 2 and 4C–E; Fig. S2). This dissociation between gene expression and activity indicates acetylene likely acts as a direct, reversible RDase inhibitor, potentially through active site binding or metal centre coordination, consistent with its known effects on metalloenzymes [56]. The specificity argues against general toxicity and validates acetylene for distinguishing OHR-dependent growth from other processes. Despite bin.2 and bin.5 each encoding nitrogenase (NifHDK), capable of reducing acetylene to ethene, no ethene formation was observed, likely due to nitrogenase inhibition by ammonium in the medium (9 mM) [41, 56]. No acetylene hydratase genes were detected, excluding acetylene as a carbon source [57, 58].

The discrepancy between rapid RDase induction within 30 min (Fig. 4C–E) and the 2-day activity lag (Fig. 1B) likely reflects time needed for translation, cofactor incorporation and/or enzyme accumulation from low initial cell density. Our single 30-min timepoint may miss complex temporal dynamics, including possible succession where different RDases dominate at different stages. Time-series transcriptomic and proteomic analyses would clarify whether the four RDase systems maintain coordinated expression or exhibit temporal niche partitioning.

**Figure 6:**
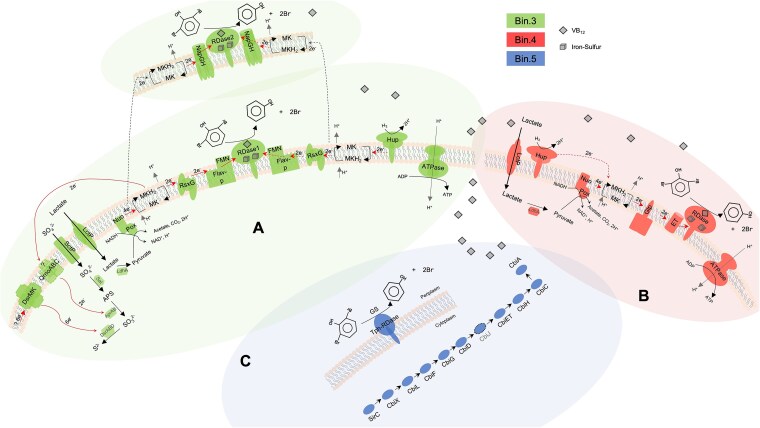
Proposed schematic overview of electron transport chains (ETCs) of organohalide respiration associated with sulfate reduction and de novo B_12_ biosynthesis. Electron transfer from lactate or H_2_ to the RDases and other proteins encoded on bin.3 to catalyze OHR and sulfate reduction (A). RDase of bin.4 follows a similar ETC pattern without sulfate reduction (B). In contrast, non-respiratory TPh-RDase of bin.5 transforms 2,6-DBP to phenol and provides the essential B_12_ for OHR to respiratory RDases (C). Color patterns used in this figure are in line with Fig. 5. MKH_2_/MK: Menaquinones; NapGH: Periplasmic nitrate reductase; Flav-p: Flavoprotein, which can bind with flavin mononucleotides (FMN); RsxG: Electron transporter, homologous with RnfG [60]; Nuo: NADH dehydrogenase; pox: Pyruvate dehydrogenase; LctP: Lactate permease; LdhA: Lactate dehydrogenase; SctP: Sulfate permease; sat: Sulfate adenylyltransferase; AprAB: APS reductase; DsrABD: Dissimilatory sulfite reductase; DsrMK: Electron transport complex function with DsrABD; QmoABC: Electron transport complex; hup: [Ni-Fe] hydrogen uptake-type hydrogenases, includes the large and small subunits; Glp: Glycerol-3-phosphate (G3P) dehydrogenase complex; ET: Ferredoxin as intermediate electron transporter; SirC-CbiA: Complete B_12_ de novo biosynthesis. Red arrows indicate the assumed electron flow, black dotted arrows indicate parallel electron flow in (A), and CbiJ as the supplementary from bin.4 (B) to bin.5 (C) to form a complete B_12_ biosynthesis pathway; “? 6e^−^”: Unknown source of deprived electrons; “? QmoABC”: Not found in genomic annotation, and could be replaced by another functionally similar complex.

Based on genomic analysis, we propose electron transport chains (ETC) coupling debromination to energy conservation. Bin.3 encodes two RDases with adjacent genes for electron transfer components: RDase1 is flanked by flavin mononucleotide (FMN)-binding flavoprotein (predicted RdhC-like function [59]) and RsxG (electron transfer from quinol pool [60]), while RDase2 is associated with NapGH quinol dehydrogenase [8, 61]. During growth on lactate, lactate and pyruvate dehydrogenases oxidize substrates to acetate, feeding electrons into the menaquinol pool for simultaneous OHR and sulfate reduction (Fig. 6A). Although the consortium lacks QmoABC typically involved in the conversion of APS to sulfite [42], alternative complexes likely fulfill this role, with Dsr proteins reducing sulfite to sulfide. Under sulfate-free conditions, fermentative lactate metabolism produces hydrogen, which is oxidized by Ni-Fe hydrogenases (HupLS) to supply electrons to RDases. Bin.4’s ETC likely functions similarly (Fig. 6B), with the encoded glycerol-3-phosphate dehydrogenase complex (GlpACB) potentially providing electrons to its RDase [62]. The TPh-RDase in bin.5, while non-respiratory, was highly expressed (Fig. 4E), suggesting active contribution to debromination.

The consortium’s B_12_-prototrophic capacity is most likely attributable to bin.5 (*Desulforhopalus*), which encodes the most complete B_12_ biosynthesis pathway (Fig. 6C). Although bin.3 possesses a partial pathway, bin.5 alone appears capable of B_12_ production at the individual population level. This metabolic self-sufficiency in the dominant population ensures B_12_ availability for the corrinoid-dependent RDases in bin.3 and bin.4, either through bin.5’s intracellular use or potential B_12_ release and uptake by consortium members. While the consortium exhibits sustained debromination in B_12_-free medium and possesses genetic capacity for B_12_ biosynthesis, we have not performed direct corrinoid measurements or quantified rate differences with B_12_ supplementation. Corrinoid profiling and dose–response experiments would provide more definitive evidence.

In conclusion, this work provides evidence for anaerobic TPh-RDase function in *Desulforhopalus*, identifies *Marinifilum* and *Ancylomarina* as candidate OHRB taxa expanding known phylogenetic diversity, and demonstrates metabolic self-sufficiency through B_12_ biosynthetic capacity enabling OHR in oligotrophic marine sediments. Future work should prioritize: (1) biochemical validation of proposed ETCs through purified RDase characterization and membrane fractionation; (2) detailed characterization of the *Desulforhopalus* TPh-RDase under anoxic conditions to elucidate catalytic mechanism, cofactor requirements, and potential energy coupling; (3) isolation and physiological characterization of individual strains, particularly *Marinifilum* and *Ancylomarina* representatives, enabling controlled co-culture experiments to dissect metabolic interactions; (4) elucidation of acetylene inhibition mechanism and its utility for distinguishing OHR from nitrogen fixation in low-ammonium environments; and (5) global metagenomic surveys combined with in situ measurements to assess prevalence and ecological significance of TPh-RDase-encoding anaerobes and newly identified OHRB taxa in marine biogeochemical cycling, accounting for potential differences between laboratory enrichment and natural sediment conditions. These investigations will advance fundamental understanding of microbial dehalogenation and practical applications in bioremediation of marine halogenated contaminants.

## Supplementary Material

Figure_S1_1_wrag007

Figure_S2_1_wrag007

Figure_S3_1_wrag007

Figure_S4_1_wrag007

ISMEJ-D-25-02040_Supplement_Final_wrag007

## Data Availability

The nucleotide sequencing data, metagenome assembly and metagenome-assembled genomes in this study have been deposited in the European Bioinformatics Institute under BioProject PRJEB106085.
