# Supplementary figures and images for: Organohalide respiration by a Desulforhopalus-dominated community
Source: ISME J. 2026 Jan 26;20(1):wrag007. doi: 10.1093/ismejo/wrag007 (PMC12908670; doi:10.1093/ismejo/wrag007)

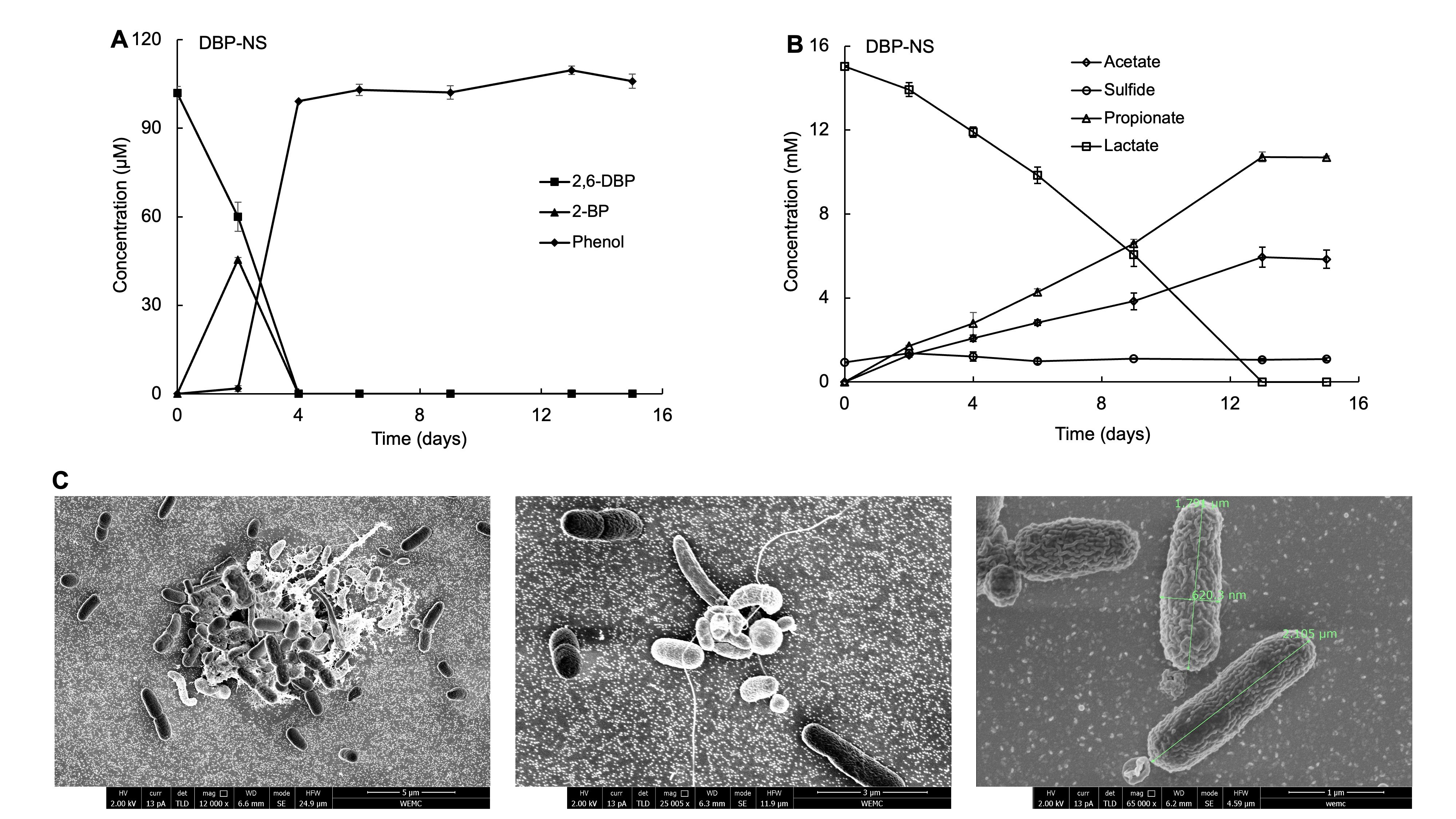

Supplement: Figure_S1_1_wrag007 [file figure_s1_1_wrag007.jpeg]

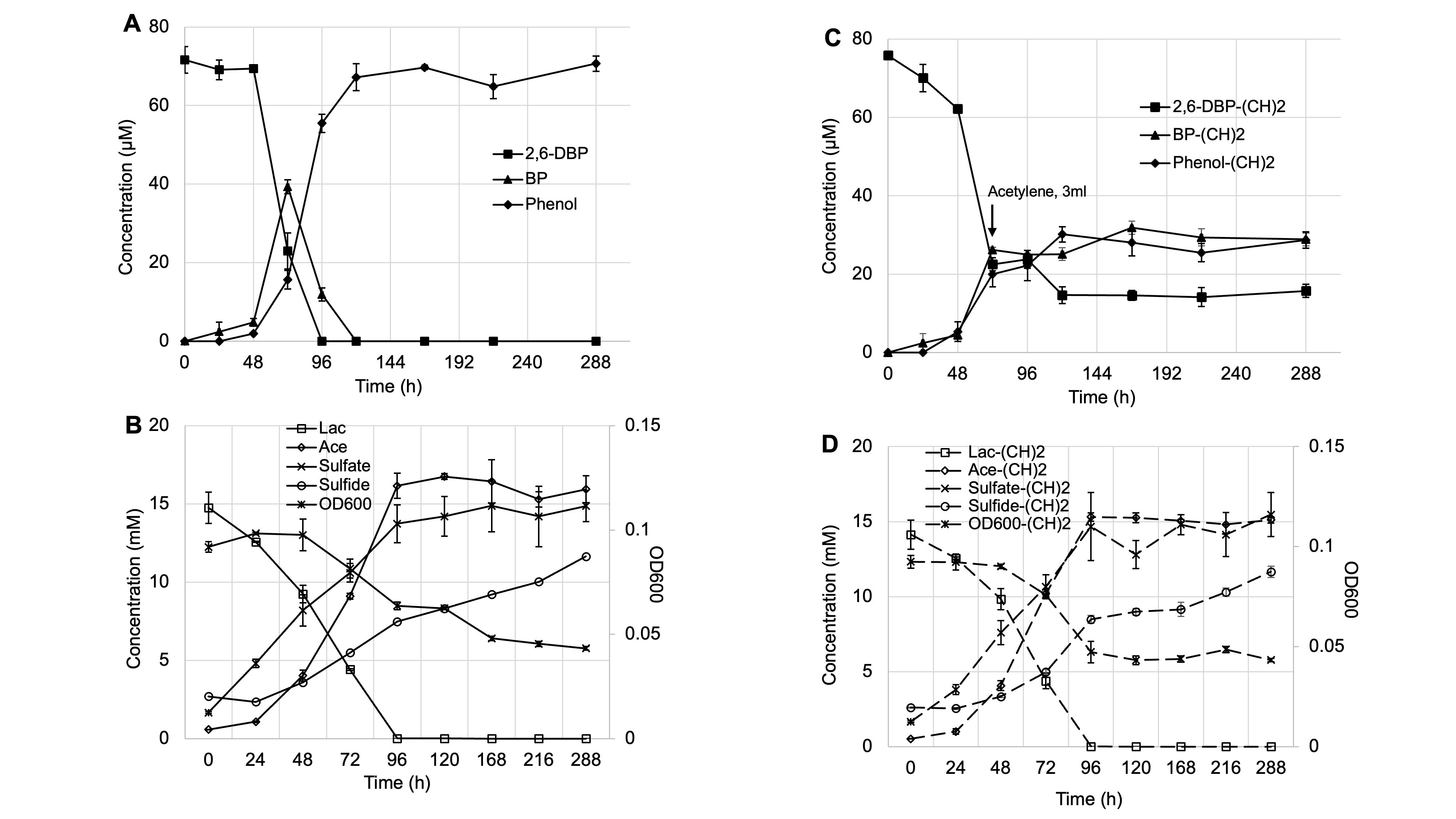

Supplement: Figure_S2_1_wrag007 [file figure_s2_1_wrag007.jpeg]

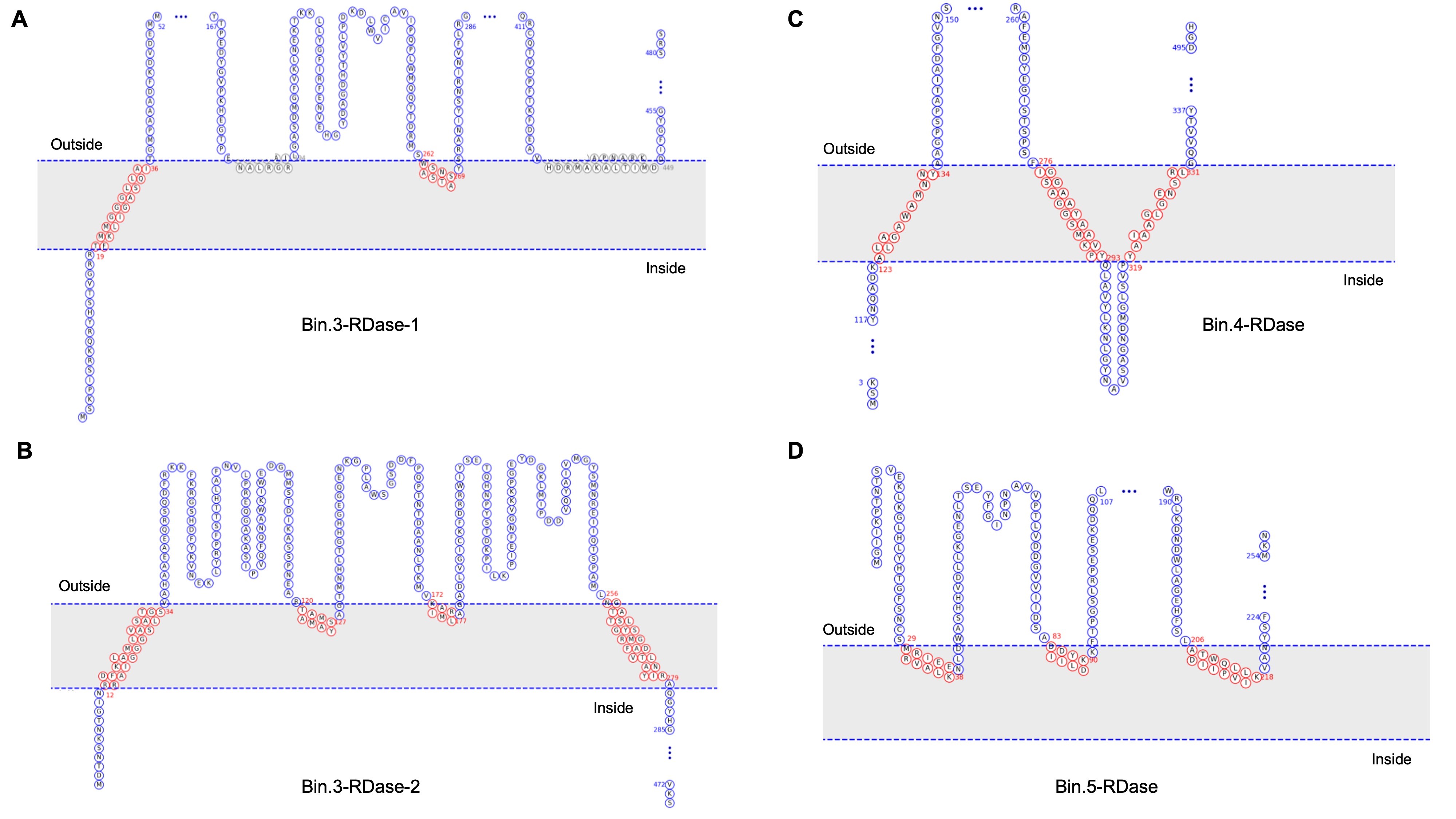

Supplement: Figure_S3_1_wrag007 [file figure_s3_1_wrag007.jpeg]

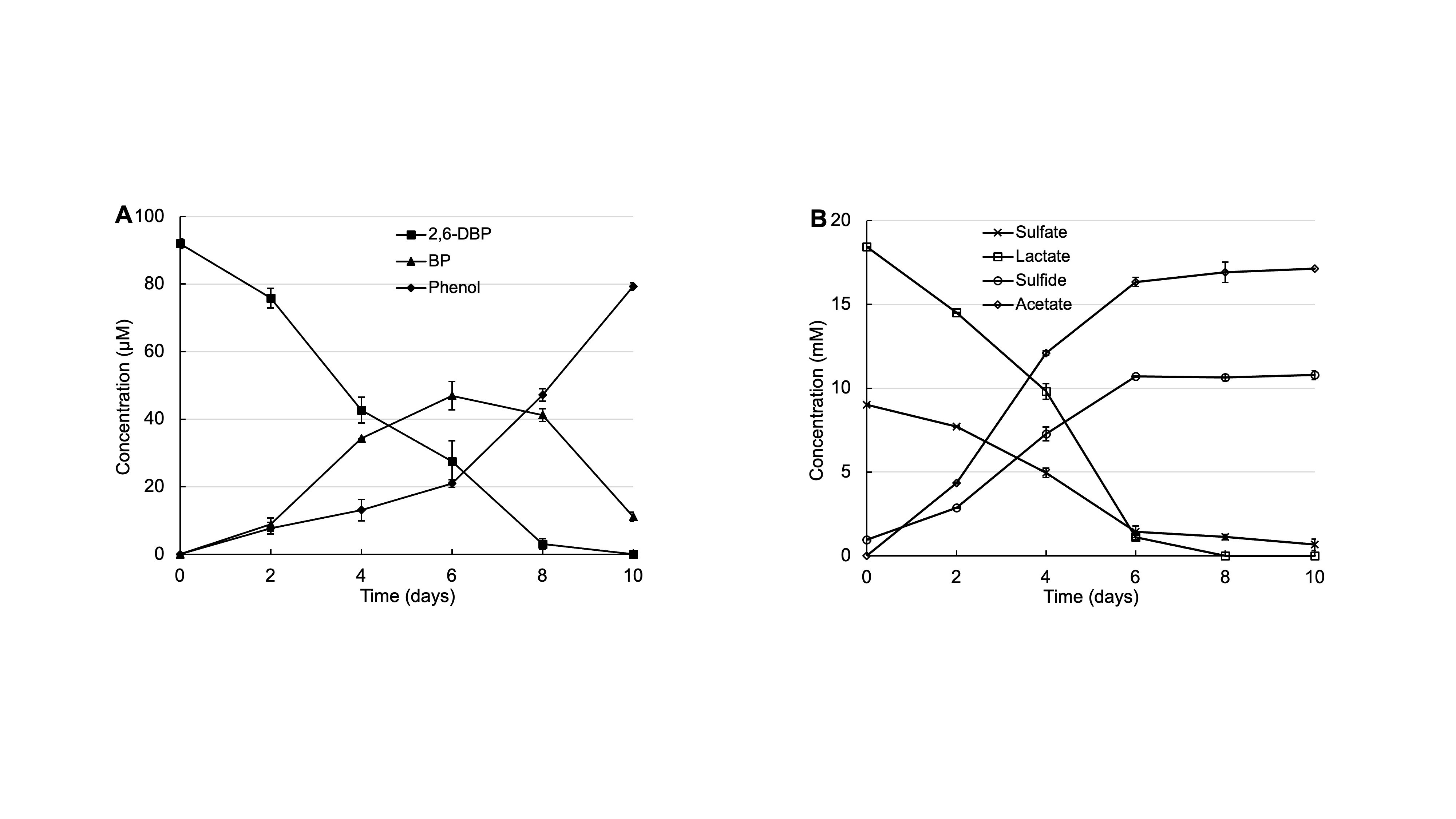

Supplement: Figure_S4_1_wrag007 [file figure_s4_1_wrag007.jpeg]
