# Supplementary material for: Organohalide respiration by a Desulforhopalus-dominated community
Source: ISME J. 2026 Jan 26;20(1):wrag007. doi: 10.1093/ismejo/wrag007 (PMC12908670; doi:10.1093/ismejo/wrag007)
Supplement: ISMEJ-D-25-02040_Supplement_Final_wrag007 [file ismej-d-25-02040_supplement_final_wrag007.docx]

**Supplementary Data**

**Organohalide respiration by a *Desulforhopalus*-dominated community**

Chen Zhang^a,b☒^, Siavash Atashgahi^a,c^, Tom N.P. Bosma^a,d^, Hauke Smidt^a☒^

^a^ Laboratory of Microbiology, Wageningen University & Research, Stippeneng 4, 6708 WE Wageningen, The Netherlands

^b^ Wenzhou Institute, University of Chinese Academy of Sciences, Wenzhou 325001, PR China

^c^ Current address: AB Mauri, Oude Kerkstraat 55, 4878 AK, Etten-Leur, The Netherlands

^d^ Current address: Deltares, Daltonlaan 600, 3484 BK, Utrecht, The Netherlands

^☒^Corresponding author 1: Laboratory of Microbiology, Wageningen University & Research, Stippeneng 4, 6708 WE Wageningen, The Netherlands. Tel: +86 18810891307; E-mail: [chen03.zhang@gmail.com](mailto:chen03.zhang@gmail.com);

^☒^Corresponding author 2: Laboratory of Microbiology, Wageningen University & Research, Stippeneng 4, 6708 WE Wageningen, The Netherlands. Tel: +31317483102; E-mail: [hauke.smidt@wur.nl](mailto:hauke.smidt@wur.nl);

**Materials and methods**

**Scanning Electron Microscopy (SEM)**

The culture was sampled for field emission scanning electron microscopy (FE-SEM). Five ml culture was sampled and incubated with glutaraldehyde (2.5%) for 20 min, and then prefixed to the cover slide coated with poly-L-lysine for 2 h. After that, the cover slide was washed three times with 0.1 M sodium cacodylate (pH 7.2) and then fixed for 1 h with 1% osmium tetroxyde in the same cacodylate buffer. Finally, the samples were dehydrated with increasing ethanol concentrations (30%, 50%, 70%, 85%, 95%, and 100% ethanol) and incubated for 10 min at each step. The final 100% ethanol step was repeated twice to ensure complete dehydration. Imaging of the sample was completed using a Magellan 400 instrument at the Wageningen Electron Microscopy Center (WEMC).

**Figure legends**

**Figure S1.** Reductive debromination of 2,6-dibromophenol (2,6-DBP) under sulfate-free conditions and scanning electron microscopy (SEM) of the consortium (related to Figure 1). 2,6-DBP was debrominated into phenol with the formation of 2-bromophenol as the intermediate (2-BP) (A). Lactate was consumed with the formation of propionate and acetate (B). The consortium was visualized by SEM at various magnifications, 12000 X (left), 25005 X (center) and 65000 X (right) (C). Three replicate bottles (n=3) were set, and the data indicate the mean ± SD. Error bars represent the SD. The rod-shaped bacterium was measured in diameter (620.3 nm) and length (1.791 – 2.105 μm) as shown in green lines and letters.

**Figure S2** Inhibition by acetylene specifically of reductive debromination of 2,6-DBP in the presence of sulfate (related to Figure 2). Reductive debromination of 2,6-DBP in addition to sulfate as the electron acceptor, and metabolite measurement with lactate as the electron donor and carbon source in the absence of acetylene (A, B), and presence of acetylene (C, D) respectively. (CH)2: acetylene; downward arrow indicates the injection of acetylene; Dashed lines represent the metabolism of lactate and sulfate after the addition of acetylene. Three replicate bottles (n=3) were set, and the data indicate the mean ± SD. Error bars represent the SD.

**Figure S3** Transmembrane configuration prediction of RDases and Tph-RDase of assembled bins (related to Figure 4), including two RDases from bin.3 (A, B); one RDase from bin.4 (C) and TPh-RDase from bin.5 (D). The prediction of transmembrane configuration of the (TPh-) RDases was achieved by MemBrain [1].

**Figure S4** Reductive debromination of 2,6-DBP without additional B12 (related to Figure 5). Reductive debromination of 2,6-DBP to phenol with bromophenol as the intermediate (A). Lactate served as the electron donor and carbon source and was consumed to form acetate, and sulfate as the electron acceptor was reduced to sulfide (B). 2,6-DBP: 2,6-dibromophenol; BP: bromophenol; The experiment was set with three replicate bottles (n=3), and the data indicate the mean ± SD. Error bars represent the SD.

**Reference**

1. Feng S-H et al. Topology prediction improvement of α-helical transmembrane proteins through helix-tail modeling and multiscale deep learning fusion. *J Mol Biol* 2020;**432**:1279–1296. https://doi.org/10.1016/j.jmb.2019.12.007
